# MagCluster: a Tool for Identification, Annotation, and Visualization of Magnetosome Gene Clusters

**DOI:** 10.1128/mra.01031-21

**Published:** 2022-01-13

**Authors:** Runjia Ji, Wensi Zhang, Yongxin Pan, Wei Lin

**Affiliations:** a Key Laboratory of Earth and Planetary Physics, Institute of Geology and Geophysics, Chinese Academy of Sciences, Beijing, China; b France-China Joint Laboratory for Evolution and Development of Magnetotactic Multicellular Organisms, Chinese Academy of Sciences, Beijing, China; c College of Earth and Planetary Sciences, University of Chinese Academy of Sciences, Beijing, China; Indiana University, Bloomington

## Abstract

Magnetosome gene clusters (MGCs), which are responsible for magnetosome biosynthesis and organization in magnetotactic bacteria (MTB), are the key to deciphering the mechanisms and evolutionary origin of magnetoreception, organelle biogenesis, and intracellular biomineralization in bacteria. Here, we report the development of MagCluster, a Python stand-alone tool for efficient exploration of MGCs from large-scale (meta)genomic data.

## ANNOUNCEMENT

The discovery of magnetotactic bacteria (MTB) has transformed our understanding of magnetoreception, organelle biogenesis, and biomineralization in the domain *Bacteria* (1–4). MTB biomineralize intracellular, membrane-bound, nano-sized magnetic crystals called magnetosomes and are characterized by their ability to sense and swim along the geomagnetic field lines (5). Genes responsible for magnetosome biosynthesis and organization are clustered together in MTB genomes, referred to as magnetosome gene clusters (MGCs) (6). Recent advances in omics-based and cultivation approaches have led to the recovery of unprecedented amounts of (meta)genomic data, sparking a need for rapid and accurate identification and comparison of various MGCs in newly reconstructed genomes. FeGenie is a hidden Markov model (HMM)-based tool that was recently developed to identify iron-related genes, including a small group of magnetosome genes, in genomes and metagenomic assemblies (7). However, the library of FeGenie lacks accessory magnetosome genes such as *mms*, *mad*, and *man* genes, and the comparison and visualization of MGCs are not supported by FeGenie.

**FIG 1:**
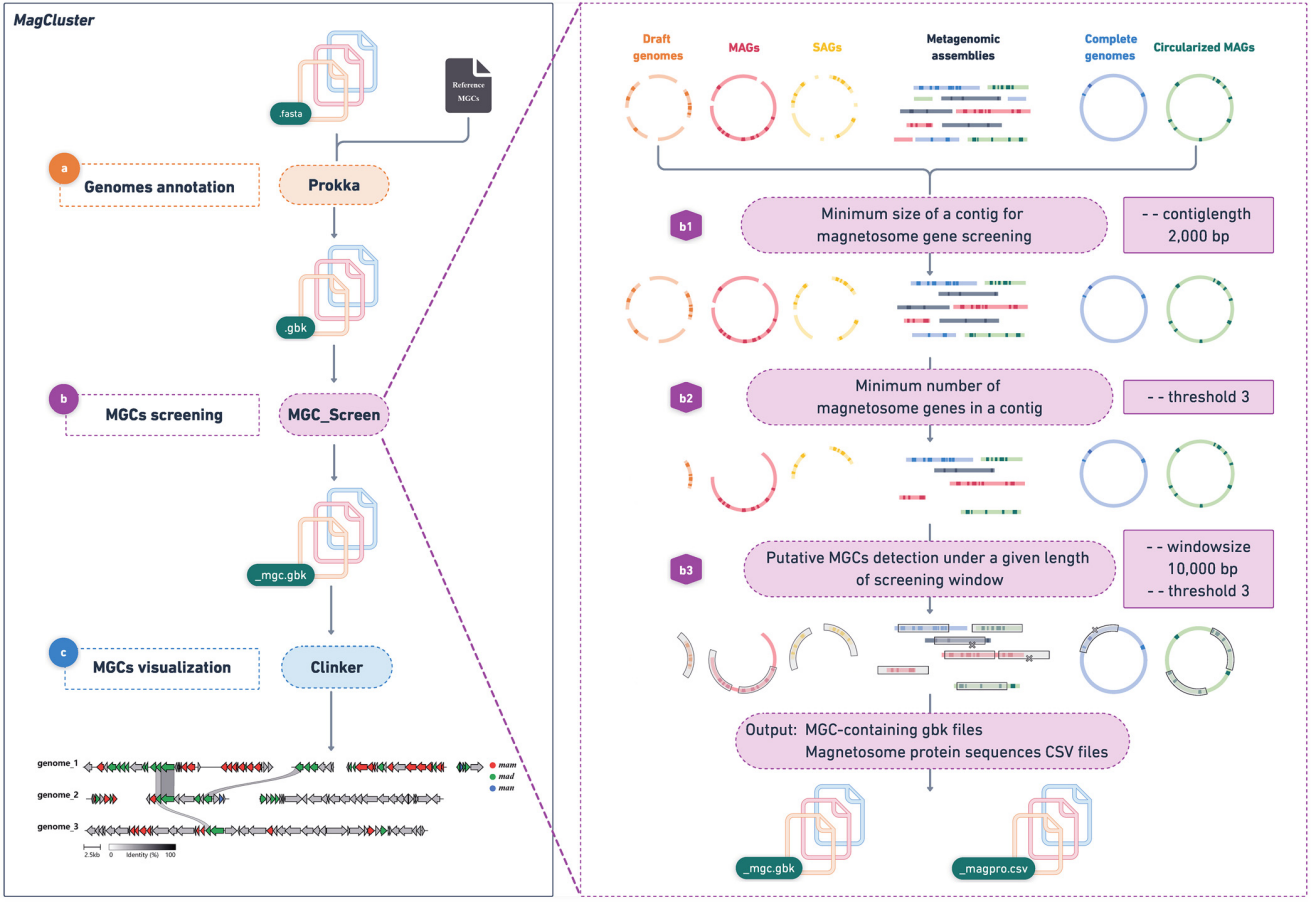
Overview of the MagCluster workflow. (a) Genomes are annotated using Prokka with a mandatory reference file of magnetosome proteins via ‐‐proteins. (b) Putative MGCs or MGC-containing contigs are retrieved by the MGC_Screen module from GenBank files generated by the annotation module. First, contigs are filtered by the contig length (‐‐contiglength) and the minimum number of magnetosome genes in a contig (‐‐threshold). Then, the length of a genomic region containing no less than the given number of magnetosome genes is checked to meet the value of ‐‐windowsize. Finally, contigs that pass all restrictions are regarded as putative MGC-containing contigs. (b1) Contigs shorter than 2,000 bp (by default) are discarded. (b2) Magnetosome genes are identified through a text-mining strategy using the keyword “magnetosome” in protein names, and contigs containing fewer than 3 (by default) magnetosome genes are discarded. (b3) Putative MGCs are screened under a 10,000-bp (by default) window, and the minimum number of magnetosome genes (3 by default) in each window size is rechecked. (c) Putative MGCs are compared and visualized using clinker. MAGs, metagenome-assembled genomes; SAGs, single amplified genomes.

Here, we present MagCluster, a tool for identification, annotation, comparison, and visualization of MGCs from large-scale (meta)genomic data. MagCluster comprises three modules (Fig. 1), (i) genome annotation with Prokka (8), (ii) MGC screening with MGC_Screen developed here, and (iii) MGC comparison and visualization with clinker (9).

For genome annotation, MagCluster provides a mandatory reference file containing a total of 192 magnetosome protein sequences, including both Fe_3_O_4_- and Fe_3_S_4_-producing proteins and both core and accessory magnetosome proteins (Mam, Mms, Mad, and Man), from seven representative MTB genomes (see https://doi.org/10.6084/m9.figshare.16863646.v3). This magnetosome protein reference file is applied with the ‐‐proteins parameter as default during genome annotation using Prokka v1.13.4 (8).

The MGC_Screen module retrieves putative MGCs or MGC-containing contigs from GenBank files generated by the genome annotation module. MGC_Screen applies a text-mining strategy for product names containing “magnetosome” to identify putative magnetosome proteins. Because magnetosome genes are clustered together in the genome, it is a useful and robust criterion to identify MGCs based on the existence of multiple magnetosome genes adjacent to each other. MGC_Screen identifies putative MGCs based on the existence of multiple magnetosome genes (‐‐threshold, 3 by default) in a given contig (‐‐contiglength, 2,000 bp by default) and a given size of the sequence screening window (‐‐windowsize, 10,000 bp by default) (Fig. 1b1 to b3). Users are advised to explore the different values of ‐‐threshold and ‐‐windowsize to achieve the best result. Note that, although MGC_Screen could efficiently identify putative MGCs, further manual review is necessary, considering the high level of genomic diversity of MGCs across different lineages.

MagCluster incorporates clinker v0.0.23 (9) to conduct the comparison and visualization of identified MGCs. An interactive HTML webpage is generated, where users can modify the MGC figure. Automatic modifications are conducted, including coloring the magnetosome genes and revising gene labels and legends.

Four MTB genomes (see https://doi.org/10.6084/m9.figshare.16864372.v2) from different taxonomic lineages were chosen to validate the effectiveness of MagCluster. MagCluster processed all four genomes and generated the MGC figure (see https://doi.org/10.6084/m9.figshare.16831012.v2) on a personal laptop using 6 cores and 8 GB of RAM, with a total runtime of 11 min 54.3 s.

In summary, MagCluster leverages the colocalization of magnetosome genes on the chromosome to identify MGCs, which are otherwise difficult to accurately identify based solely on sequences. MagCluster will facilitate future surveys of MGCs and MTB from large-scale (meta)genomic data.

### Data availability.

MagCluster can be downloaded from Python Package Index (PyPI) and Bioconda under the GNU General Public License v3.0. MagCluster is available on GitHub (https://github.com/runjiaji/magcluster) and Gitee (https://gitee.com/runjiaji/magcluster).
